# SUPPORT Tools for evidence-informed health Policymaking (STP) 2: Improving how your organisation supports the use of research evidence to inform policymaking

**DOI:** 10.1186/1478-4505-7-S1-S2

**Published:** 2009-12-16

**Authors:** Andrew D Oxman, Per Olav Vandvik, John N Lavis, Atle Fretheim, Simon Lewin

**Affiliations:** 1 Norwegian Knowledge Centre for the Health Services, P.O. Box 7004, St. Olavs plass, N-0130 Oslo, Norway; 2 Norwegian Knowledge Centre for the Health Services, P.O. Box 7004, St. Olavs plass, N-0130 Oslo, Norway and Department of Medicine, Innlandet Hospital Health Authority, Gjøvik, Norway; 3 Centre for Health Economics and Policy Analysis, Department of Clinical Epidemiology and Biostatistics, and Department of Political Science, McMaster University, 1200 Main St. West, HSC-2D3, Hamilton, ON, Canada, L8N 3Z5; 4 Norwegian Knowledge Centre for the Health Services, P.O. Box 7004, St. Olavs plass, N-0130 Oslo, Norway; Section for International Health, Institute of General Practice and Community Medicine, Faculty of Medicine, University of Oslo, Norway; 5 Norwegian Knowledge Centre for the Health Services, P.O. Box 7004, St. Olavs plass, N-0130 Oslo, Norway; Health Systems Research Unit, Medical Research Council of South Africa

## Abstract

This article is part of a series written for people responsible for making decisions about health policies and programmes and for those who support these decision makers.

In this article, we address ways of organising efforts to support evidence-informed health policymaking. Efforts to link research to action may include a range of activities related to the production of research that is both highly relevant to – and appropriately synthesised for – policymakers. Such activities may include a mix of efforts used to link research to action, as well as the evaluation of such efforts. Little is known about how best to organise the range of activity options available and, until recently, there have been relatively few organisations responsible for supporting the use of research evidence in developing health policy. We suggest five questions that can help guide considerations of how to improve organisational arrangements to support the use of research evidence to inform health policy decision making. These are: 1. What is the capacity of your organisation to use research evidence to inform decision making? 2. What strategies should be used to ensure collaboration between policymakers, researchers and stakeholders? 3. What strategies should be used to ensure independence as well as the effective management of conflicts of interest? 4. What strategies should be used to ensure the use of systematic and transparent methods for accessing, appraising and using research evidence? 5. What strategies should be used to ensure adequate capacity to employ these methods?

## About STP

*This article is part of a series written for people responsible for making decisions about health policies and programmes and for those who support these decision makers. The series is intended to help such people ensure that their decisions are well-informed by the best available research evidence. The SUPPORT tools and the ways in which they can be used are described in more detail in the Introduction to this series *[1]. *A glossary for the entire series is attached to each article (see Additional File *1). *Links to Spanish, Portuguese, French and Chinese translations of this series can be found on the SUPPORT website http://www.support-collaboration.org). Feedback about how to improve the tools in this series is welcome and should be sent to: STP@nokc.no*.

## Scenario

There is a new Minister of Health in the Ministry where you work. One of the goals of the new Minister is to improve the capacity of the Ministry to use research evidence to inform decisions about how the health system is organised, financed and governed. You have been asked to put forward a proposal for strategies to improve how the Ministry supports its use of evidence to inform policy decisions.

## Background

In this article, we present five questions that policymakers and those who support them could ask when considering how to improve support for the use of research evidence to inform health policy decisions. Such questions could, for instance, be asked by any of the people in the scenario outlined above.

A number of theories have been proposed to explain the role of research evidence in policymaking. In addition, common wisdom about how to improve the appropriate use of research evidence is abundant. However, empirical evidence to support such ideas is difficult to find [2]. While increasing numbers of studies are being undertaken in low- and middle-income countries [3-8] most evidence still comes from interview studies in high-income countries [9,10]. Systematic reviews of these studies suggest that [9,10]:

• Interaction between researchers and policymakers increases the likelihood of research being used by policymakers

• Good timing and timely research increase (and poor timing or lack of timeliness decrease) the likelihood of research being used by policymakers

• When policymakers have negative attitudes towards research evidence, the likelihood of research being used by them decreases

• When policymakers lack relevant skills and expertise, the likelihood of research being used by them decreases

• Policy networks and trust in researchers increase the likelihood of research being used by policymakers, and

• A lack of perceived relevance, the use of jargon, and the production of publications aimed at a scholarly audience are all factors that decrease the likelihood of research being used by policymakers

Activities aimed at improving the use of research evidence to inform policy have been referred to in various ways. These terms include: knowledge translation, knowledge transfer, knowledge exchange, research utilisation, implementation, diffusion, and dissemination [11]. Considerable confusion and misunderstanding exists about the definition and scope of these concepts, and the literature related to these issues is diverse and widely dispersed [12]. Several frameworks have been proposed as ways to organise these approaches and thus improve the use of research evidence by policymakers [11-20]. These frameworks have overlapping purposes and concepts.

One of these frameworks focuses on assessing country-level efforts to link research to action. This framework provides an inventory of a range of activities that can be considered when developing organisational arrangements to support the use of research evidence to inform health policy decisions [20]. It includes four elements: the general climate for research use, the production of research that is both highly relevant to – and appropriately synthesised for – policymakers, the mix of efforts used to link research to action, and the *evaluation* of efforts to link research to action. Within this framework, efforts to link research to action are categorised in four clusters of activities. These are: *push efforts* (efforts to communicate research findings which may include, for example, the tailoring of messages by researchers according to policymaker needs), *efforts to facilitate user pull* (such as rapid-response units to meet policymaker needs for research evidence), *user pull* (efforts to facilitate research use, such as efforts to train policymakers in how to access research evidence), and *exchange efforts* (partnerships between researchers and policymakers in which relevant questions are jointly asked and answered).

Little is known about how best to organise such a range of activities and, until recently, relatively few organisations were responsible for supporting the use of research evidence in developing health policy [21,22]. The questions that we propose in this article focus on the lessons learned from the experience of organisations engaged in activities to support evidence-informed health policymaking [21]. The evidence from which these lessons were drawn was collected from a survey of 176 organisations, followed by telephone interviews with 25 of these, and site visits to eight. The lessons are:

• Establish strong links between policymakers and researchers, and involve stakeholders in the work undertaken

• Be independent and manage conflicts of interest among those involved in the work

• Use appropriate methods and be transparent in the work

• Collaborate with other organisations

• Start small, have a clear audience and scope, and address important questions

• Build capacity among those working in the organisation

• Be attentive to implementation considerations even if implementation is not a remit

## Questions to consider

**Figure 1:**
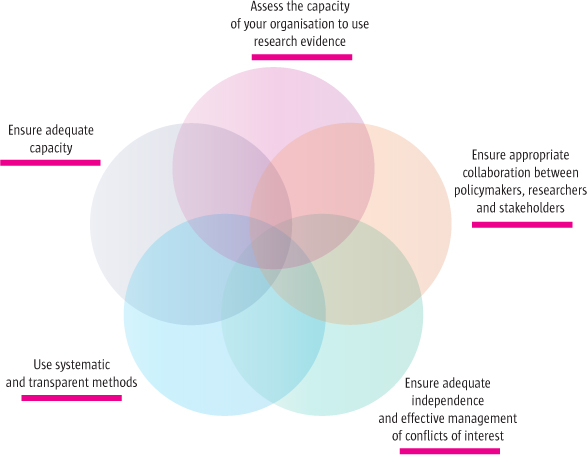
Strategies to improve how support for evidence-informed health policymaking is organised

Drawing on the above lessons, we suggest five questions that can be asked when considering how to improve support for the use of research evidence to inform health policy decisions. These questions address key strategies to improve how support for evidence-informed health policymaking is organised, as illustrated in Figure 1. They do not address broader questions about government policymaking processes and how these can be designed to promote the use of evidence. The questions are:

1. What is the capacity of your organisation to use research evidence to inform decision making?

2. What strategies should be used to ensure collaboration between policymakers, researchers and stakeholders?

3. What strategies should be used to ensure independence as well as the effective management of conflicts of interest?

4. What strategies should be used to ensure the use of systematic and transparent methods for accessing, appraising and using research evidence?

5. What strategies should be used to ensure adequate capacity to employ these methods?

### 1. What is the capacity of your organisation to use research evidence to inform decision making?

In order for organisations to improve the degree to which their decisions are well-informed by research evidence, sufficient capacity is needed to recognise the need for research evidence. This is necessary for acquiring research when it is needed, critically appraising it, using it to inform decisions, and measuring the impacts of policies and programmes that are implemented [20,23-27]. Capacities in these different areas vary widely both in governmental and non-governmental organisations [21,28]. A first step in the process of improving organisational capacity is therefore the assessment of an organisation’s current capacity.

There are a number of validated instruments for measuring the competence of individuals to practice evidence-based medicine e.g.[29-31]. However, in this article, our focus is on *organisational* capacity rather than the competence of individuals. The Canadian Health Services Research Foundation (CHSRF) has developed a self-assessment tool for healthcare organisations to assist in identifying ways in which research can be gathered and used, as well as potential ways in which this can be improved [23,24,32,33]. This tool includes four key areas for the assessment of research use: the acquisition, assessment, adaptation and application of evidence. Lavis and colleagues have proposed a framework for assessing country-level efforts to link research to action [20]. Their framework includes a number of areas not covered by the CHSRF tool [23]. These include the extent to which the general environment supports the linking of research to action, the production of research, efforts to communicate research findings (so-called ‘push’ strategies), and efforts to facilitate the use of research findings (so-called ‘user pull’ factors).

The self-assessment tool that we present in Additional File 2 draws on both of these frameworks, and the CHSRF tool in particular. It addresses the key steps needed to ensure the appropriate use of research evidence to inform decisions related to health policies and programmes. This tool is intended to help organisations assess and improve their capacity to use research evidence. It has not been formally tested. Instead, it has evolved through iterative revisions based on workshops involving a variety of groups.

Additional File 2 is a ‘scorecard’ intended to provide the basis for discussion and for reaching agreement about the priorities and strategies needed for improvement. Often people in the same organisation have divergent perceptions of how well the organisation is doing. This is illustrated in Additional File 3, which summarises the application of a scorecard to the assessment of an organisation’s performance. Identifying and discussing these discrepancies can help to develop a shared vision and a plan of action. The scorecard shown in Additional File 2 can be applied across departments in a large organisation (as highlighted in the illustrative example shown in Additional File 3), as well as within a department, or a combination of both. The scorecard can also be used to monitor how well an organisation is doing in its efforts to improve its use of research evidence.

### 2. What strategies should be used to ensure collaboration between policymakers, researchers and stakeholders?

Many organisations that support the use of research evidence in policymaking commonly involve policymakers in the selection of topics and the services undertaken. Personal communication between policymakers and researchers has been found to be particularly important, both by policymakers and those who support their use of research evidence [21]. Organisations that support evidence-informed policymaking view their close links with policymakers as a strength [21]. However, this strength brings with it a related challenge: the need to manage conflicts of interest that can emerge in any close relationship between researchers and policymakers.

Strategies that can help to ensure collaboration between policymakers and researchers include:

• Locating those who support the use of research by policymakers (by accessing, appraising and summarising evidence) within or close to those organisations responsible for policymaking

• Involving policymakers on an advisory board or steering committee in instances when organisations are located outside government or policymaking organisations

• Formal agreements linking academic organisations to policymaking organisations

• Using trusted individuals as ‘knowledge brokers’ to build relationships among researchers and policymakers [34]

• Involving policymakers in research processes such as the preparation of policy briefs [35]

• Involving researchers in policy-informing processes such as policy dialogues [36]

• Skill development programmes for both policymakers and researchers [37-39] including exchanges where researchers are seconded to a policymaking organisation and policymakers are seconded to a research organisation

An illustration of the need to manage potential tensions between policymakers and researchers who are working together is provided in Table 1.

**Table 1:** A case study of the need to manage tensions between policymakers and researchers in a long-term collaboration

Since the early 1990s, policymakers in the provincial government of the Free State in South Africa have worked closely with researchers on health and health policy-related topics, including the monitoring and evaluation of antiretroviral (ARV) therapy rollouts [21]. The evidence from these studies has exposed major deficiencies in the ARV rollout, and concerns have been raised that if the research findings become too critical, the privileged data access offered to researchers, and the collaboration offered on evaluations, may simply end. This has led to tensions in the relationship between the researchers and the provincial Health Department with both sides being very direct about these concerns. While acknowledging that it is challenging to manage the tensions, both the policymakers and the researchers are committed to learning how to manage this kind of conflict. From the Health Department’s perspective, this is essential in order to evaluate and improve the services delivered by the provincial government. From the researchers’ perspective, this is motivated by “a feeling that you are doing research that is actually relevant and addressing actual needs as opposed to just driving publications” [21].

Organisations that support the use of research evidence in policymaking also frequently cite the involvement of stakeholders as a key strength [21]. Stakeholder organisations include, for example, patient organisations, community groups, coalitions, advocacy groups, faith-based organisations, charities or voluntary organisations, professional associations, trade unions and business associations [40].

However, managing stakeholder involvement can be both challenging and demanding. There is a paucity of evidence comparing alternative ways of involving stakeholders in policymaking or research processes including [41]:

• The degree of involvement (consultation or collaboration)

• Different forums for communication (e.g. committee membership, permanent panels, town meetings, interviews, written consultation)

• Different methods for recruiting stakeholders (e.g. targeted personal invitations, advertisements, or the use of mass media)

• Different ways of training and supporting consumers or other stakeholders to ensure effective involvement

• Different degrees of financial support to facilitate the involvement of consumers or other stakeholders

There is a range of different types of collaboration that may be appropriate for different stakeholders. For some groups, ongoing interaction may be more useful than involving them directly in policymaking (e.g. groups that have an interest in one aspect of a policy, such as professional regulatory issues). For other groups, it may be desirable to keep them at arms length (e.g. pharmaceutical companies with a vested interest in a policy decision). For certain groups, it may be justifiable to exclude them completely from deliberations (e.g. tobacco companies that have falsified research results on the harmful effects of tobacco).

Strategies that can help to ensure appropriate levels of stakeholder involvement are similar to those highlighted above for ensuring collaboration between policymakers and researchers. These may include, for example, the involvement of stakeholders on an advisory board or steering committee, in research processes, and in policymaking processes. They may also include consultation with stakeholder groups, the use of skill-development programmes for stakeholders [42-44], and the communication of evidence to the wider public via the mass media [45].

An example of the use of extensive strategies for involving stakeholders by a public agency is provided in Table 2.

**Table 2:** An example of stakeholder involvement in healthcare decisions: the National Institute for Health and Clinical Excellence (NICE)

Few organisations have sought to integrate stakeholders (especially patients and their caregivers) more thoroughly than the National Institute for Health and Clinical Excellence (NICE) in England and Wales [50]. NICE has created effective strategies to involve stakeholder groups including [50-52]: • A programme within the Institute with dedicated staff responsible for patient and public involvement • The identification and recruitment of stakeholders, including lay people, to NICE’s independent advisory committees • The provision of training and support to lay people on NICE’s committees • The registration of stakeholder groups, which are then routinely consulted electronically and through meetings • The involvement of stakeholders throughout the development of guidance and decisions from topic selection to reviews of draft guidance, through to consultation and active participation on committees • Systematic and transparent responses to stakeholders’ comments on drafts • The development and dissemination of lay versions of NICE’s guidance, versions for key stakeholder groups, and mass media briefings, as well as versions for clinicians and managers, and • The involvement of stakeholders in guidance implementation
NICE’s experience suggests that the involvement of stakeholders in healthcare decision making is possible and can work well, but requires strong commitment and specific arrangements. It can also be costly. Although NICE’s investment in stakeholder involvement is widely valued, it is uncertain whether the right stakeholders are involved, both in terms of which stakeholder groups engage in the process and in terms of the extent to which the individuals who become involved appropriately represent various stakeholders. It is also uncertain whether the strategies they use are as efficient as they could be – in other words, whether the resources invested in those processes represent good value for money [51]. There are also concerns about the growing burden of managing stakeholder input. Although the number of submissions from stakeholders has been increasing, involvement at the individual level within stakeholder organisations may be less than desired.

### 3. What strategies should be used to ensure independence as well as the effective management of conflicts of interest?

Independence is the most commonly cited strength of organisations that support the use of research evidence in policymaking [21]. Conversely, conflicts of interest are seen as a key weakness. Financial and intellectual independence and freedom from government and industry influence are viewed as the key strengths of such organisations. But these need to be balanced against the desirability of arrangements that can ensure collaboration between policymakers and researchers. Independence is, of course, relative. No organisation is entirely independent.

Mutually agreed processes and methods are essential in order to manage possible competing tensions arising from the demands of both collaboration and independence. They are also important as ways to ensure the systematic and transparent access and appraisal of evidence as an input into the policymaking process.

Conflicting interests frequently underlie tensions arising between policymakers, researchers and other stakeholders. Although there is little empirical evidence to guide arrangements for managing conflicts of interest, the key options that warrant consideration include [46]:

• Specific, detailed, structured disclosure forms that solicit as much information as possible about the nature and extent of competing interests. Minimal or open-ended formats for disclosure forms are likely to be uninformative

• Explicit criteria to make decisions easier about whether a disclosed interest constitutes a conflict of interest

• A range of management strategies to address disclosed conflicts of interest, ranging from the public disclosure of conflicts associated with each meeting as a minimum prerequisite, through to the recusal of conflicted individuals as the most extreme measure

• A standard policy requiring all financial ties to be made public (e.g. that they be recorded in meeting minutes), may reduce the number of problematic cases

• A standing committee to review all financial disclosure statements prior to the commencement of committee meetings or hearings, to make management recommendations when necessary, and which can help to ensure that conflict of interest policies are enforced

Organisational arrangements should ensure responsiveness to the information needs of policymakers. At the same time, it is important to ensure independence with respect to the methods used to access, appraise and summarise research evidence. Arrangements to ensure that independence is maintained may include:

• Financial arrangements that minimise the risk of inappropriate influence on what evidence is summarised, or how it is summarised

• Management arrangements, including the involvement of independent stakeholders in advisory boards or steering groups

• Mechanisms for managing disputes such as independent arbitrators or appeal processes, particularly for governmental agencies that fund the work and for industry

• Ensuring that decision making is transparent in terms of how evidence is accessed, appraised, summarised and publicly reported

### 4. What strategies should be used to ensure the use of systematic and transparent methods for accessing, appraising and using research evidence?

The majority of organisations supporting the use of research evidence in policymaking use systematic reviews [21]. In addition to their independence, such organisations commonly state that their use of systematic and transparent methods (sometimes they are referred to as “being evidence-based”) is one of their key strengths. However, organisations that support governments to use research evidence in the development of health policies and programmes are less likely to have guidelines describing the methods they use. They are also less likely to conduct or use systematic reviews relative to organisations that produce health technology assessments (HTAs) or clinical practice guidelines. In addition, using systematic and transparent methods brings a related challenge: the time-consuming nature of using more rigorous methods. As a consequence, many organisations, particularly HTA agencies, have attempted to develop more rapid methods that are “quick but clean enough” [47].

Given that evidence-informed health policymaking is characterised by the use of systematic and transparent methods to access and appraise evidence as an input into the policymaking process, it therefore follows that the use of agreed-upon methods for doing this is key for any organisational arrangement to support evidence-informed policymaking. Such methods should be described in easily accessible documents. Moreover, although organisational arrangements are likely to vary widely, a great deal of commonality in the methods that are used is likely, as is the case for clinical practice guidelines, for example [48]. Thus, in addition to helping to ensure the use of agreed-upon methods, accessible manuals that describe these methods can also benefit other organisations with similar interests.

Stakeholders who feel that they have lost out as the result of a particular decision are still likely to challenge the methods used if there is a substantial amount at stake, irrespective of the rigour and transparency applied. Nonetheless, the use of agreed-upon methods that are described in easily accessible form can make it easier to respond to such challenges.

An illustration of efforts to ensure the use of systematic and transparent methods to develop recommendations and policies is provided in Table 3.

**Table 3:** An example of ensuring the use of systematic and transparent methods in an international organisation

The World Health Organization (WHO) has had guidelines for guidelines since 2003, emphasising the use of systematic reviews for the evidence of effects, processes that allow for the explicit incorporation of other types of information (including values), and evidence-informed dissemination and implementation strategies. However, until 2007 systematic reviews were rarely used for developing recommendations [53]. Instead, processes usually relied heavily on experts in a particular specialty, rather than representatives of those who have to live with the consequences of those recommendations, or experts in particular methodological areas. To address these problems and to ensure the use of systematic and transparent methods, WHO has taken a number of actions, based on a review of its own work and the methods used by others [21,26,40,53-55]. These actions include: • Revising and updating a manual describing the methods that are to be used, which is updated and revised based on both WHO’s experience and new developments • Establishing a committee with a mandate to review and approve plans for developing recommendations prior to initiating the work, and recommendations prior to their publication • Developing checklists for *assessing* recommendations and plans for *developing* recommendations based on the manual • Establishing a secretariat and a network to provide training and support to implement the methods described in the manual, and • Monitoring and evaluating the impacts of these arrangements to ensure the use of systematic and transparent methods

### 5. What strategies should be used to ensure adequate capacity to employ these methods?

The most commonly cited weakness of organisations that support the use of research evidence in policymaking are a lack of financial and human resources. How adequate funding for supporting the use of research evidence can be ensured is a major challenge, particularly in low- and middle-income countries. Partly, this may be because this function falls between two stools – it is typically not funded by research funders, or by those interested in strengthening policymaking. Identifying appropriate sources of funding is critical to developing and sustaining adequate capacity for supporting evidence-informed health policymaking.

Three of the key messages that emerged from a review of these organisations relate to ensuring adequate capacity [21,49]:

• Collaborate with other organisations, both informally and formally, to learn from their experience in order to avoid the unnecessary duplication of efforts, to draw on their capacity, *and* to help build capacity (see Table 4 for examples of international collaboration)

**Table 4:** Examples of collaborations with other organisations

The following are examples of international collaborations that help to build capacity and support for the use of research evidence in health policymaking:
**EVIPNet (the Evidence Informed Policy Network)** – initiated by the World Health Organization and the Ministries of Health in 25 countries, its aim is to promote the use of research evidence in health policy formulation in order to strengthen health systems [22,56]. At the country level, EVIPNet takes the form of partnerships between policymakers, researchers and civil society and focuses on facilitating the use of research evidence. Launched in 2005, EVIPNet now supports activities in Africa, Asia and the Americas.
**Region of East Africa Community Health (REACH) policy initiative** – established within the East African Community (EAC) (Kenya, Tanzania and Uganda, with the recent addition of Rwanda and Burundi) to bridge the gap between evidence and health policy and practice [57]. Its mission is to access, synthesise, package and communicate evidence required for policy and practice and to influence policy-relevant research agendas for improved population health and health equity in each of the member countries.
**Reforming States Group (RSG)** – since 1991, leaders in health policy from the legislative and executive branches of state government, with the financial support and staff collaboration of the Milbank Memorial Fund, have shared their experiences and have worked on practical solutions to shared healthcare problems. They have focused increasingly on the use of research evidence to inform health policy decisions [38,39,58]. The RSG now also includes members outside the United States of America. The Center for Evidence-based Policy, which works with RSG members, was established in 2003 by former Oregon Governor, John Kitzhaber, to address public policy challenges by identifying and applying the best available evidence through self-governing communities of interest [59].
**Cochrane Collaboration** – a global network whose aim is to improve healthcare decision making through the preparation and updating of systematic reviews of the effects of healthcare interventions. The Cochrane Collaboration ensures that these reviews are made accessible. See http://cochrane.org/

• Build capacity among those working in the organisation through training, making the best use of available staff (numbers are often limited), and actions aimed at retaining skilled staff, and

• Start small, have a clear scope, and address important questions in order to ensure that available resources are focused on areas where they are needed most

As noted above, another strategy that many organisations identified was the use of more rapid methods that are rigorous but less resource-intensive – especially those that would result in a reduction in the time required of skilled staff.

## Conclusion

A scorecard, such as the one shown in Additional File 2, can be used to assess the capacity of an organisation to support its use of research evidence. This can provide a useful basis for discussion and for establishing consensus about an organisation’s strengths, weaknesses, priorities and the strategies necessary for improvement. Although people in the same organisation often have divergent views about how well it is performing, identifying and discussing these discrepancies can help to develop a shared vision and plan of action. This may be achieved, for example, by sharing information within or across different sections or levels within the organisation, clarifying what different sections of the organisation can or should be doing, addressing misunderstandings, resolving communication problems or identifying information that is needed to resolve disagreements.

There is limited evidence regarding the effects of different strategies to improve how support for evidence-informed health policymaking is organised. Organisational arrangements should logically be tailored to address specific aims and circumstances. Nonetheless, a number of lessons can be drawn from the experience of organisations around the world. Reflection on the questions discussed in this article can help policymakers and those who support them to improve organisational arrangements supporting the use of research evidence to inform health policy decisions.

## Resources

### Useful documents and further reading

- Moynihan R, Oxman AD, Lavis JN, Paulsen E. Evidence-Informed Health Policy: Using Research to Make Health Systems Healthier. Rapport Nr 1-2008. Oslo: Nasjonalt kunnskapssenter for helsetjenesten, 2008. http://www.nokc.no/Publikasjoner/469.cms

- Alliance for Health Policy and Systems Research. Strengthening health systems: the role and promise of policy and systems research. Geneva: Alliance for Health Policy and Systems Research, 2004. http://www.who.int/alliance-hpsr/resources/Strengthening_complet.pdf

- Lavis JN, Lomas J, Hamid M, Sewankambo NK. Assessing country-level efforts to link research to action. Bull World Health Organ 2007; 84:620-8. http://www.scielosp.org/scielo.php?pid=S0042-96862006000800013&script=sci_arttext&tlng=en

- EUnetHTA Work Package 8. EUnetHTA Handbook on Health Technology Assessment Capacity Building. Barcelona: Catalan Agency for Health Technology Assessment and Research. Catalan Health Service. Department of Health Autonomous Government of Catalonia; 2008. http://www.gencat.cat/salut/depsan/units/aatrm/pdf/eunethta_wp8_hb_hta_capacity_building.pdf

- Thornhill J, Judd M, Clements D. CHSRF Knowledge Transfer: (Re)introducing the self-assessment tool that is helping decision-makers assess their organization’s capacity to use research. Healthc Q 2008; 12:22-4. http://www.longwoods.com/product.php?productid=20410

### Links to websites

- Evidence-Informed Policy Network (EVIPNet): http://www.evipnet.org/php/index.php – EVIPNet promotes the systematic use of health research evidence in policymaking. Focusing on low- and middle-income countries, EVIPNet promotes partnerships at the country level between policymakers, researchers and civil society in order to facilitate both policy development and policy implementation through the use of the best scientific evidence available. EVIPNet comprises networks that bring together country-level teams, which are coordinated at both regional and global levels.

- Alliance for Health Systems Policy and Research: http://www.who.int/alliance-hpsr/en/ –The Alliance for Health Policy and Systems Research is an international collaboration based in the WHO, Geneva. It has its origins in the recommendations of the 1996 report of WHO’s Ad Hoc Committee on Health Research which identified a lack of health policy and systems research as a key problem impeding the improvement of health outcomes in low- and middle-income countries. It aims to promote the generation and use of health policy and systems research as a means to improve the health systems of developing countries.

- Canadian Health Services Research Foundation: http://www.chsrf.ca – The Foundation brings researchers and decision makers together to create and apply knowledge to improve health services for Canadians. It is an independent, not-for-profit corporation, established with endowed funds from the federal government and its agencies.

## Competing interests

The authors declare that they have no competing interests.

## Authors’ contributions

ADO prepared the first draft of this article. POV, JNL, AF and SL contributed to drafting and revising it.

## Supplementary Material

Additional file 1 Glossary Click here for file

Additional file 2 Click here for file

Additional file 3 Click here for file
