# Supplementary material for: SUPPORT Tools for evidence-informed health Policymaking (STP) 2: Improving how your organisation supports the use of research evidence to inform policymaking
Source: Health Res Policy Syst. 2009 Dec 16;7(Suppl 1):S2. doi: 10.1186/1478-4505-7-S1-S2 (PMC3271829; doi:10.1186/1478-4505-7-S1-S2)
Supplement: Additional file 2 [file 1478-4505-7-S1-S2-S2.doc]

**Additional File 2. Self-assessment of organisational capacity to support the use of research evidence to inform decisions**

**Name of organisation, department or unit: _____________________________________________**

| 1. **Do your organisational culture and values support the use of research evidence to inform decisions?** | | | | | | | | | | | | | | | | | | |
| --- | --- | --- | --- | --- | --- | --- | --- | --- | --- | --- | --- | --- | --- | --- | --- | --- | --- | --- |
| **Don’t  know**  **0** | | **Strongly disagree**  **1** | **Disagree**  **2** | **Neither agree nor disagree**  **3** | **Agree**  **4** | | | | | **Strongly agree**  **5** | | | | | | | | |
|  | Our **mission or other key organisational documents** support evidence-informed decisions | | | | | 0 | | 1 | | | 2 | | 3 | | 4 | | 5 | |
|  | **Leadership** in the organisation supports evidence-informed decisions | | | | | 0 | | 1 | | | 2 | | 3 | | 4 | | 5 | |
|  | We are active members in **networks** that support evidence-informed policymaking or actively follow the developments and the products of relevant networks | | | | | 0 | | 1 | | | 2 | | 3 | | 4 | | 5 | |
|  | We have **regular meetings** where highly relevant research evidence is discussed in relationship to decisions | | | | | 0 | | 1 | | | 2 | | 3 | | 4 | | 5 | |
|  | Our organisation has **committed resources to ensure that research evidence is used** to inform decisions | | | | | 0 | | 1 | | | 2 | | 3 | | 4 | | 5 | |
|  | **Overall**, our **organisational culture and values** support the **use of research evidence** to inform decisions | | | | | 0 | | 1 | | | 2 | | 3 | | 4 | | 5 | |
| **Comments about how your organisation is doing:**  **Additional information that is needed to assess how your organisation is doing or to resolve disagreements:**  **Priorities for improvements (suggested actions to address weaknesses or build on strengths):** | | | | | | | | | | | | | | | | | | |
| 1. **Does your organisation do a good job of setting priorities for obtaining research evidence to inform decisions?** | | | | | | | | | | | | | | | | | | |
| **Don’t  know**  **0** | | **Strongly disagree**  **1** | **Disagree**  **2** | **Neither agree nor disagree**  **3** | **Agree**  **4** | | | | | **Strongly agree**  **5** | | | | | | | | |
|  | We have **explicit criteria** for setting priorities for obtaining research evidence | | | | | | 0 | | 1 | | | 2 | | 3 | | 4 | | 5 |
|  | An **appropriate mix of people** with relevant types of expertise, responsibilities and interests make decisions about priorities for obtaining research | | | | | | 0 | | 1 | | | 2 | | 3 | | 4 | | 5 |
|  | We have an **appropriate process** for setting priorities for obtaining research evidence dynamically | | | | | | 0 | | 1 | | | 2 | | 3 | | 4 | | 5 |
|  | We have **appropriate priorities for obtaining research evidence** | | | | | | 0 | | 1 | | | 2 | | 3 | | 4 | | 5 |
|  | **Overall**, our organisation does a good job of **setting priorities** for obtaining research evidence to inform decisions | | | | | | 0 | | 1 | | | 2 | | 3 | | 4 | | 5 |
| **Comments about how your organisation is doing:**  **Additional information that is needed to assess how your organisation is doing or to resolve disagreements:**  **Priorities for improvements (suggested actions to address weaknesses or build on strengths):** | | | | | | | | | | | | | | | | | | |

| 1. **Does your organisation do a good job of obtaining research evidence to inform decisions?** | | | | | | | | | | | | |
| --- | --- | --- | --- | --- | --- | --- | --- | --- | --- | --- | --- | --- |
| **Don’t  know**  **0** | | **Strongly disagree**  **1** | **Disagree**  **2** | **Neither agree nor disagree**  **3** | **Agree**  **4** | | | **Strongly agree**  **5** | | | | |
|  | We have **skilled staff** to search for and retrieve research evidence | | | | | 0 | 1 | | 2 | 3 | 4 | 5 |
|  | Our staff have **enough time, incentive and resources** or **arrangements with external experts** to find and obtain research evidence | | | | | 0 | 1 | | 2 | 3 | 4 | 5 |
|  | We have good **access to databases** such as PubMed and The Cochrane Library **and publications that report relevant research** | | | | | 0 | 1 | | 2 | 3 | 4 | 5 |
|  | We have **good access to national, provincial or local evidence** that we need to inform decisions (e.g. routinely collected data, surveys, one-off studies) | | | | | 0 | 1 | | 2 | 3 | 4 | 5 |
|  | **Overall**, our organisation does a good job of **obtaining** research evidence to inform priority decisions | | | | | 0 | 1 | | 2 | 3 | 4 | 5 |
| **Comments about how your organisation is doing:**  **Additional information that is needed to assess how your organisation is doing or to resolve disagreements:**  **Priorities for improvements (suggested actions to address weaknesses or build on strengths):** | | | | | | | | | | | | |
| 1. **Does your organisation do a good job of assessing the quality and applicability of research evidence and interpreting the results to inform priority decisions?** | | | | | | | | | | | | |
| **Don’t  know**  **0** | | **Strongly disagree**  **1** | **Disagree**  **2** | **Neither agree nor disagree**  **3** | **Agree**  **4** | | | **Strongly agree**  **5** | | | | |
|  | We have **skilled staff** to evaluate the quality and applicability of research evidence and interpret the results | | | | | 0 | 1 | | 2 | 3 | 4 | 5 |
|  | Our staff have **enough time, incentive and resources** to evaluate the quality and applicability of research evidence and interpret the results | | | | | 0 | 1 | | 2 | 3 | 4 | 5 |
|  | We have **arrangements with external experts** to evaluate the quality and applicability of research evidence and interpret the results | | | | | 0 | 1 | | 2 | 3 | 4 | 5 |
|  | **Overall**, our organisation does a good job of assessing the quality and applicability of research evidence and interpreting the results to inform priority decisions | | | | | 0 | 1 | | 2 | 3 | 4 | 5 |
| **Comments about how your organisation is doing:**  **Additional information that is needed to assess how your organisation is doing or to resolve disagreements:**  **Priorities for improvements (suggested actions to address weaknesses or build on strengths):** | | | | | | | | | | | | |

| 1. **Does your organisation do a good job of using research evidence to inform recommendations and decisions?** | | | | | | | | | | | | |
| --- | --- | --- | --- | --- | --- | --- | --- | --- | --- | --- | --- | --- |
| **Don’t  know**  **0** | | **Strongly disagree**  **1** | **Disagree**  **2** | **Neither agree nor disagree**  **3** | **Agree**  **4** | | | **Strongly agree**  **5** | | | | |
|  | Our staff have **sufficient time, expertise and incentive** to ensure appropriate use of research evidence to inform **recommendations and decisions** | | | | | 0 | 1 | | 2 | 3 | 4 | 5 |
|  | Staff and appropriate stakeholders **know how and when they can contribute research evidence to inform decisions and how that information will be used** | | | | | 0 | 1 | | 2 | 3 | 4 | 5 |
|  | Our organisation ensures **that appropriate stakeholders are involved** in decision making and that they have access to relevant research evidence | | | | | 0 | 1 | | 2 | 3 | 4 | 5 |
|  | **What evidence was used and how** it was used is **transparent in our decisions** | | | | | 0 | 1 | | 2 | 3 | 4 | 5 |
|  | **Overall**, our organisation does a good job of **using research** evidence to inform **recommendations and decisions** | | | | | 0 | 1 | | 2 | 3 | 4 | 5 |
| **Comments about how your organisation is doing:**  **Additional information that is needed to assess how your organisation is doing or to resolve disagreements:**  **Priorities for improvements (suggested actions to address weaknesses or build on strengths):** | | | | | | | | | | | | |
| 1. **Does your organisation do a good job of monitoring and evaluating policies and programmes?** | | | | | | | | | | | | |
| **Don’t  know**  **0** | | **Strongly disagree**  **1** | **Disagree**  **2** | **Neither agree nor disagree**  **3** | **Agree**  **4** | | | **Strongly agree**  **5** | | | | |
|  | We **routinely consider the need for monitoring and evaluation** | | | | | 0 | 1 | | 2 | 3 | 4 | 5 |
|  | Our staff have **enough expertise or adequate arrangements with external experts** for monitoring and evaluation | | | | | 0 | 1 | | 2 | 3 | 4 | 5 |
|  | Our staff have the **incentive and resources to conduct or commission monitoring and evaluation** | | | | | 0 | 1 | | 2 | 3 | 4 | 5 |
|  | Our organisation ensures **that appropriate stakeholders are involved** in decisions about **monitoring and evaluation** | | | | | 0 | 1 | | 2 | 3 | 4 | 5 |
|  | **Overall**, our organisation does a good job of **monitoring and evaluation** of policies and programmes | | | | | 0 | 1 | | 2 | 3 | 4 | 5 |
| **Comments about how your organisation is doing:**  **Additional information that is needed to assess how your organisation is doing or to resolve disagreements:**  **Priorities for improvements (suggested actions to address weaknesses or build on strengths):** | | | | | | | | | | | | |

| 1. **Does your organisation do a good job of supporting continuing professional development that addresses important topics and is evidence-based?** | | | | | | | | | | | | |
| --- | --- | --- | --- | --- | --- | --- | --- | --- | --- | --- | --- | --- |
| **Don’t  know**  **0** | | **Strongly disagree**  **1** | **Disagree**  **2** | **Neither agree nor disagree**  **3** | **Agree**  **4** | | | **Strongly agree**  **5** | | | | |
|  | Our staff have **enough time for continuing professional development** | | | | | 0 | 1 | | 2 | 3 | 4 | 5 |
|  | We have routines to ensure that our staff continue to develop **appropriate skills for obtaining, appraising and applying research evidence** | | | | | 0 | 1 | | 2 | 3 | 4 | 5 |
|  | Our staff **prioritise continuing professional development activities that are “evidence-based”** (i.e. with content that is based on research evidence and using continuing professional development methods that are based on research evidence) | | | | | 0 | 1 | | 2 | 3 | 4 | 5 |
|  | We have **appropriate routines for prioritising internal professional continuing development activities** that accommodate the needs of both new and long-term staff | | | | | 0 | 1 | | 2 | 3 | 4 | 5 |
|  | We have **appropriate routines for deciding whether to support participation in external continuing professional development activities** that accommodate the needs of both new and long-term staff | | | | | 0 | 1 | | 2 | 3 | 4 | 5 |
|  | **Overall**, our organisation does a good job of **supporting continuing professional** development that addresses important topics and is evidence-based | | | | | 0 | 1 | | 2 | 3 | 4 | 5 |
| **Comments about how your organisation is doing:**  **Additional information that is needed to assess how your organisation is doing or to resolve disagreements:**  **Priorities for improvements (suggested actions to address weaknesses or build on strengths):** | | | | | | | | | | | | |
