# Supplementary material for: SUPPORT Tools for evidence-informed health Policymaking (STP) 2: Improving how your organisation supports the use of research evidence to inform policymaking
Source: Health Res Policy Syst. 2009 Dec 16;7(Suppl 1):S2. doi: 10.1186/1478-4505-7-S1-S2 (PMC3271829; doi:10.1186/1478-4505-7-S1-S2)
Supplement: Additional file 3 [file 1478-4505-7-S1-S2-S3.doc]

**Additional File 3. An example of an organisation’s self-assessment of its capacity to use research evidence to inform decision making**

**Organisational culture and values**

At a workshop, a group of people from different departments within an international organisation assessed the capacity of their organisation to use research evidence using a scorecard similar to the one shown in Table 1. None of the people involved were in leadership positions. Many people in the group had divergent viewpoints. Participants stated that they were unsure how well the organisation was performing in a number of areas, and perceived that there were variations in performance between different departments. There was a perception amongst the attendees that the organisational culture and values of the organisation supported the use of research evidence based on key documents and linkages to international networks. Support from leadership varied, as did the frequency of meetings and the availability of resources. The workshop group agreed that organising regular meetings focused on highly relevant research and its usefulness to the organisation could help to improve the culture of the organisation with regard to its use of research evidence. Specific strategies for organising this were discussed. The group concluded that it would be important for the organisation’s leadership to be involved in this process of organisational self‑assessment.

**Setting priorities for obtaining research to inform decisions**

The group was unsure about the use of explicit criteria for setting priorities in their organisation. They agreed generally that priorities in the organisation were set, at least broadly, by the board, which included an appropriate mix of people. This process, they felt, was largely a political one and that while it was transparent, it was not systematic. Most of the group felt that this system was appropriate. In some cases priority-setting within the organisation’s departments was seen as ad hoc. It was felt that it could be improved by having more structured discussions involving all those affected and by having clearer criteria for deciding on priorities within the broader priorities set by the board.

**Obtaining research evidence**

The group had divergent perceptions regarding their access to research evidence. These variations were shaped by their different geographical locations within the organisation as well as by differences between their departments. They also reflected the differing degrees of awareness amongst people of the services at their disposal. There was agreement that there were considerable constraints due to low staffing levels in the library, and that although there was good access to databases, there were varying degrees of difficulty with regard to accessing publications. Several strategies for improving access to research evidence were discussed, including the provision of training to improve awareness of available resources and developing searching skills. Discussions topics also included ways in which access to publications could be improved using channels already available, and capitalising on links with academic institutions.

**Critically appraising research evidence**

The group agreed that although there were pockets of strength within the organisation and although there was good access to external experts, staff did not always have adequate skills. It was agreed that a substantial proportion of the staff could benefit from workshops to help them develop these skills. Several strategies for organising training were discussed, including incorporating the training into routine meetings where relevant evidence was discussed, as well as internal and external workshops, and online training.

Members of the group were aware of important shortcomings within the organisation’s recommendations and policies. Plans for improvements included implementing standards for the development and reporting of recommendations and policies, through the establishment of a central mechanism. This mechanism would approve plans before work was started on developing recommendations or policies, and before approval was given for any final recommendations or policies. Training and the improved use of external methodological and topic experts were also recommended.

**Monitoring and evaluating the impacts of decisions**

The group did not assess how well their organisation monitors and evaluates the impacts of its policies and programmes. However, as with many governmental and non-governmental organisations, monitoring and evaluation were major organisational challenges due to limited resources and because of uncertainty about the actual impacts of most programmes and policies. The organisation did not routinely consider the need for monitoring and evaluation and had limited expertise in this area. Strategies to improve monitoring and evaluation for any organisation like this might include: building consideration of evaluation into routine processes for approving policies and programmes, using training or recruitment to increase the level of expertise within the organisation, better co-ordination with partner organisations, and making impact evaluation mandatory for policies and programmes that meet explicit criteria.

**Professional development**

As already noted, the group identified several needs within their organisation that could be addressed through continuing education. They also identified a variety of strategies that could meet those needs. They did not feel that they were in a position to set these priorities, or to address the challenges of addressing the needs of both new and long-term staff. However, they decided that this was an important issue for them to take back to their organisation. They also noted that they would suggest in their workshop report that those in mid-level leadership positions in their organisation should undertake their own assessment, and consider how to make more effective use of the resources available within the organisation to ensure continuing professional development.
